# A new surgical procedure for synchronous esophageal squamous cell carcinoma and gastric adenocarcinoma: Case report: three cases reports

**DOI:** 10.1097/MD.0000000000014725

**Published:** 2019-03-01

**Authors:** Yunpeng Zhao, Bo Cong

**Affiliations:** Department of Thoracic Surgery, The Second Hospital of Shandong University, Jinan, China.

**Keywords:** esophagus cancer, gastric cancer, gastroepiploic artery, synchronous multiple primary neoplasms, thoracoscopic surgery

## Abstract

**Rationale:**

The stomach is always used to reconstruct the upper digestive tract for esophageal cancer operation. However, problems arise when the esophageal cancer and gastric cancer present at the same time. No medical literature mentioned about this surgical procedure till now.

**Patient concerns:**

Majority of the patients had the sensation of obstruction when swallowing because of the esophageal tumor. Gastric adenocarcinoma was found when gastroscopy was performed.

**Diagnosis:**

Synchronous esophageal squamous cell carcinoma and gastric adenocarcinoma were confirmed by biopsy pathology.

**Interventions:**

We describe the new technique as: distal gastrectomy preserving the gastroepiploic vessels, Roux-en-Y gastrojejunostomy and thoracoscopic Ivor Lewis esophagectomy with chest anastomosis.

**Outcomes:**

Three patients accepted the surgery and recovered well without any complications. The patients did not undergo any postoperative adjuvant therapy and was doing well without any recurrence till date (23 months, 12 months, 6 months separately).

**Lessons:**

This procedure was less invasive and easier to perform for synchronous early-stage gastric cardiac cancer and middle or lower third thoracic esophageal cancer. We recommend the indication as: esophageal tumor was located at least 27 cm away from the incisor teeth (for R0 resection during chest anastomosis, be sure no superior mediastinal lymph nodes metastasis were found preoperation), gastric tumor was located in the distal portion of the gastric tube and evaluated for clinical stage IA.

## 1 Introduction

Synchronous esophageal squamous cell carcinoma and gastric adenocarcinoma were trending more in the recent years.^[1,2]^ The prognosis of double primary cancer of esophagus and stomach remained controversial when compared with isolated esophageal cancer or gastric cancer.^[3,4]^ There is no doubt that operation remains an important treatment for early-stage lesion, but some previous reports suggest chemo-radiotherapy or endoscopic resection.^[2,5]^ Esophageal squamous cell carcinoma associated with gastric adenocarcinoma requires more investigation as they are associated with stomach for reconstruction of the upper gastrointestinal tract.

Here, we introduced a new surgical method for patients with early stage gastric cardiac cancer and middle or lower third thoracic esophageal cancer which has never been reported till date.

## 2 Methods

### 2.1 Ethical approval and patient consent

The present study was approved by the ethics committee of the Second Hospital of Shandong University [KYLL-2018(CL)P-0010]. Informed written consent was obtained from the patient for publication of this case report and accompanying images.

### 2.2 Surgical technique

**Figure 1:**
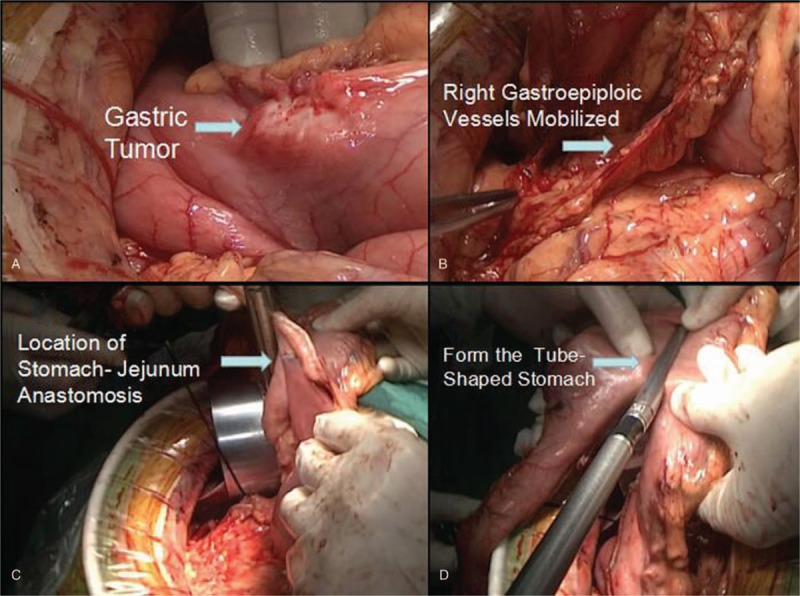
(A) Explored the gastric lesion. (B) Mobilized the right gastroepiploic vessels. (C and D) Performed the tube-shaped stomach formation after Roux-en-Y gastrojejunostomy.

*Abdominal part*: Patients were placed in supine position, and then intravenous combined general anesthesia for the insertion of double lumen endotracheal intubation was used. The gastric lesion with upper median laparotomy was explored. The greater curvature of the stomach was mobilized, the infrapyloric lymph nodes were dissected, and the branches of right gastroepiploic vessels were carefully mobilized and divided. The resection range of stomach was determined to finalize the length of the mobilized right gastroepiploic vessels. The left gastroepiploic vessels and short gastric vessels were divided. The lesser omentum was opened, the right gastric artery was cut off and the suprapyloric nodes were dissected. The lymph nodes were moved away around the esophagogastric junction and the left gastric artery. The left gastric artery was properly dealt and the nodes around the common hepatic artery, splenic artery, and celiac artery along the upper edge of the pancreas were dissected. Gastrointestinal anastomosis stapler was used to cut off the duodenal bulb, jejunum-jejunum end to side anastomosis according to Roux-en-Y gastrojejunostomy was performed. The jejunal segment prepared for anastomosis with remnant stomach was arranged to lie through the transverse mesocolon to guarantee the lifting height of the stomach. Selectively an incision on the stomach was made to accomplish the stomach-jejunum anastomosis, the tube-shaped stomach formation was performed while resecting the distal part, including the incision for anastomosis. A needle jejunostomy tube was placed on the distal side of the jejunum-jejunum anastomosis with a distance of at least 20 cm. The defect of mesojejunum and the mesocolon was sutured, and we should make sure that the residual stomach and the right gastroepiploic vessels are on the right direction (Fig. 1)

**Figure 2:**
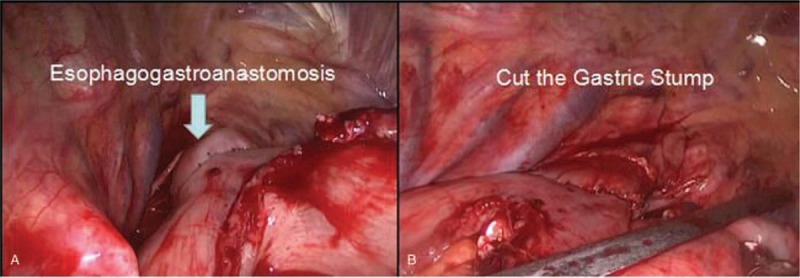
(A) Esophagogastroanastomosis. (B) The endo-GIA was used to cut the gastric stump. Endo-GIA: endo-gastrointestinalanastomosis.

*Thoracic Part*: The patient was turned to the left half of the prone position, and 4 trocars were used in the intercostal spaces of the right thoracic cavity. Artificial penumothorax was performed. The esophagus and the tumor were mobilized, making better for en bloc of periesophageal lymph nodes, subcarinal lymph nodes, left and right recurrent laryngeal nerve lymph nodes, trachea, and bronchus lymph nodes. A wound protector was placed when expanding the incision in the 4th intercostal space, the anesthesiologist was asked to change to single lung ventilation. The location of anastomosis above the level of the arch of the azygos vein was selected, the purse line around the esophagus was sutured, and the circular stapler anvil was inserted into the esophagus. The attached stomach was pulled up, and a circular stapler was used for anastomosis and the nasogastric tube was placed. The gastric stump was cut by endo- gastrointestinalanastomosis (endo-GIA) (Fig. 2).

## 3 Case reports

### 3.1 Case 1

For each case, information about the patient (age/sex/presenting complaints), diagnostic investigation and staging, the intervention, outcome and follow-up to be mentioned.

A 68-year-old asymptomatic man was referred to our hospital for evaluation because of an irregularity in the esophagus was detected during a medical examination. Esophageal squamous cell carcinoma was located at 27 to 30 cm away from the incisor teeth, the gastric adenocarcinoma was located on the lesser curvature side of sinuses ventriculi.

No surrounding tissue invasion, and no enlargement of lymph node were observed in Contrast-enhanced Computed Tomography.

*Operation*: The gastric lesion via the upper median laparotomy was explored. The right gastroepiploic artery and vein were mobilized after the excision range was ensured. The distal stomach was removed, the tube-shaped stomach was prepared, and the upper digestive tract was reconstructed using Roux-en-Y gastrojejunostomy. The patient was then turned to the left half prone position and accomplished the thoracoscopic esophagectomy and endoscopic chest anastomosis. The operation lasted for 7 hours with a bleeding volume of 200 mL, and no blood was transfused. T1bN0M0, stage IA for gastric cancer and T1bN0M0, stage IB for esophageal cancer were observed. The patient did not undergo any postoperative adjuvant therapy and the patient was doing well without any recurrence till now (23 months).

### 3.2 Case 2

A 63-year-old man had the complaints of sensation of obstruction when swallowing for 6 months. Esophageal adenosquamous cancer was located 37 to 40 cm away from the incisor teeth. The gastric mucinous adenocarcinoma was located on the big curvature above the sinuses ventriculi.

*Operation*: Surgical procedure was similar to patient 1. However, there were 2 differences: tube-shaped stomach was formed before reconstruction of the upper digestive tract for patient 1, and the procedure was performed in reverse order for patient 2; Jejunum went through the posterior pathway of the transverse colon in patient 1 and crossed the anterior wall of the transverse colon in patient 2. T3N0M0, stage IIA for esophageal cancer and T1bN0M0, stage IA for gastric cancer were observed. The patient did not undergo any postoperative adjuvant therapy and the patient enjoyed normal life without any recurrence till date (12 months).

### 3.3 Case 3

Male, 61 years old, complaints of sensation of obstruction when swallowing for 1 month. The patient's operation was performed on May 5, 2017. Anesthesia and surgical procedure was similar to the other patients described above. T3N0M0, stage IIA for esophageal cancer and T1bN0M0, stage IA for gastric cancer were observed. The patient did not undergo any postoperative adjuvant therapy and the patient enjoyed normal life without any recurrence till date (6 months).

## 4 Discussion

The surgical indications and approach of multiple primary carcinoma (MPC) depends on the clinical stage, tumor location, tumor size and surgeons’ experience. Nguyen et al^[6]^ elaborated a technique of minimally invasive Ivor Lewis esophagogastrectomy and laparoscopic colonic interposition using the right colon for gastric cardia cancer involving the gastric body and distal esophagus. Honda et al^[7]^ reported the combination of thoracoscopic esophagectomy, laparoscopic total gastrectomy, and laparoscopy assisted colon reconstruction for thoracic lower esophagus carcinoma and gastric adenocarcinoma in the middle third of the stomach. Both the operations discussed above were complicated and required the surgeons’ rich experience in performing the technique. Nguyen et al^[8]^ reported that thoracoscopic Ivor Lewis esophagectomy was performed after Roux-en-Y gastric bypass for obesity without distal stomach resection. Tumor in the middle third of thoracic esophagus with antral tumor in the patient was reported^[9]^ in the year of 2002; however, they both were advanced tumors, and had even multiple hepatic metastases. Subtotal gastrectomy was performed and Malafaia tube was inserted to solve the problem of obstruction.

**Figure 3:**
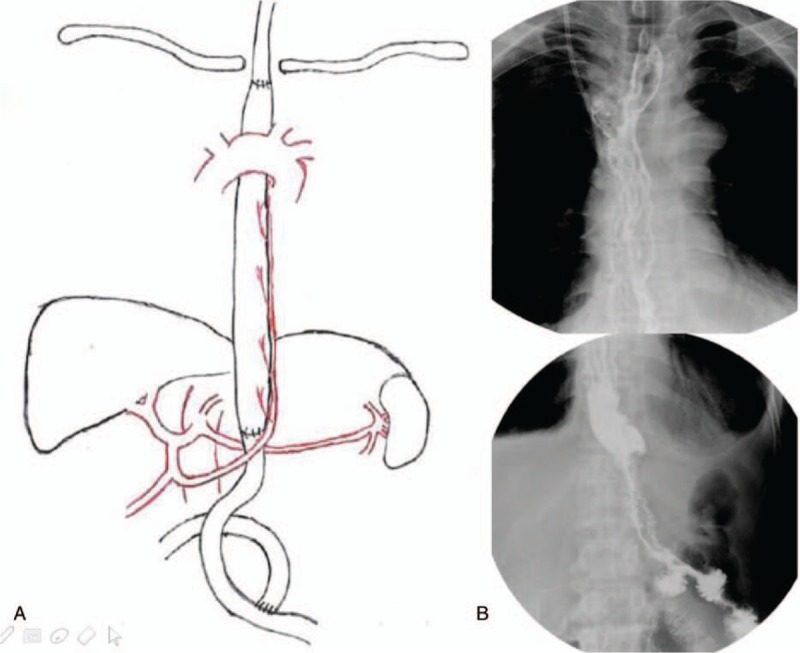
(A) Reconstruction method. (B) Postoperative gastrointestinal tract radiography.

We designed 3 schemes to deal with the synchronous esophageal carcinoma (the middle or lower third) and the gastric antral carcinoma, including the use of stomach, colon or jejunum separately. The operation was more complicated and involved higher risk for the latter 2 ways and could only be considered for individual cases. Motoyama et al^[10]^ described distal gastrectomy for preserving the right gastroepiploic vessels in gastric cancer patients after subtotal esophagectomy, and provided us crucial information on how to use the stomach for reconstruction. We discussed the procedure during preoperative conference and successfully performed it (Fig. 3). Connective tissue should be preserved to protect the right gastroepiploic vessels, and as proposed by Motoyama,^[10]^ it may be somewhat less appropriate for advanced gastric cancer due to the principle of entire dissection of lymph nodes.

To sum up, distal gastrectomy with Roux-en-Y gastrojejunostomy by preserving the right gastroepiploic vessels, combined with thoracoscopic Ivor Lewis esophagectomy was accepted for synchronous esophageal squamous cell carcinoma and gastric adenocarcinoma. We recommend the indication as: esophageal tumor was located at least 27 cm away from the incisor teeth (for R0 resection during chest anastomosis), gastric tumor was located in the distal portion of the gastric tube and evaluated for clinical stage IA. The surgical procedure remained safe and relatively easier to perform after adequate learning and training.

## Acknowledgments

The authors would like to thank Dr Ling Li from the Cheeloo Hospital of Shandong University for her picture drawing.

## Author contributions

Bo Cong designed the surgery; Yunpeng Zhao assisted to accomplish the surgery and wrote the paper.

**Conceptualization:** Bo Cong.

**Data curation:** Yunpeng Zhao.

**Writing – original draft:** Yunpeng Zhao.

**Writing – review & editing:** Yunpeng Zhao.

ORCID number: Yunpeng Zhao (https://orcid.org/0000-0002-4966-222X).

Bo Cong (https://orcid.org/0000-0002-7232-5790).
